# Triggered metabolism of adenosine triphosphate as an explanation for the chemical heterogeneity of heterotopic ossification

**DOI:** 10.1038/s42004-023-01015-z

**Published:** 2023-10-19

**Authors:** Cong Sui, Thomas E. Robinson, Richard L. Williams, Neil M. Eisenstein, Liam M. Grover

**Affiliations:** Healthcare Technologies Institute, School of Chemical Engineering, University of Birmingham, Birmingham, B15 2TT UK

**Keywords:** Biomineralization, Bioinorganic chemistry, Bioinspired materials, Mechanism of action

## Abstract

Heterotopic ossification (HO), the pathological formation of bone in soft tissues, is a debilitating condition, as well as one of the few instances of de novo bone formation in adults. Chemical mapping of HO tissue showed distinct islands of calcium phosphate within phosphate-deficient, calcium-rich regions, suggesting a transition to apatitic bone mineral from a non-phosphatic precursor. The transition of amorphous calcium carbonate (ACC), a generally suggested bone-mineral precursor, in physiological conditions was thus investigated. Here, we show that adenosine triphosphate (ATP), present in high amounts in forming bone, stabilised ACC for weeks in physiological conditions and that enzymatic degradation of ATP triggered rapid crystallisation into apatite, through an amorphous calcium phosphate phase. It is suggested that this localised enzymatic degradation could explain the chemical heterogeneity seen in HO and may also represent a pathway to physiological bone mineralisation.

## Abstract (web summary)

Heterotopic ossification (HO) is the pathological formation of bone in soft tissues involving hydroxyapatite (HA) formation in vivo, however, the exact mechanism of HA formation remains debated. Here, the authors demonstrate the chemical mapping of human HO and the transformation of amorphous calcium carbonate in physiological conditions and reveal the role of ATP in bone mineralisation.

## Introduction

Heterotopic ossification (HO) is the pathological deposition of bone in non-skeletal sites, which can occur following high-energy trauma, nervous system injury, burns, skeletal surgery, and hereditary conditions^1^. While appearing disordered and growing polyaxially at the macro-scale, the microstructure of HO is nearly identical to that of skeletal bone^2^. Therefore, in addition to researchers aiming to prevent and treat HO, this condition is of great interest to those aiming to stimulate bone formation, a key aim in regenerative medicine^3–6^. Further, HO can be a useful model to study bone development, as it represents de novo bone formation in humans, excised and available at multiple time points, that can then be characterised and studied in detail more easily than skeletal bone^7^.

Despite decades of dedicated research on the subject, there is still much debate in the bone community on the exact progression from ionic calcium and phosphate to highly structured calcium-deficient hydroxyapatite (HA) in vivo^8^. Popular explanations include the direct formation of HA, perhaps structured by the organic scaffold that forms prior to mineralisation, or HA formation within the specific environment inside osseous cells or extracellular vesicles, which is then deposited onto the scaffolds^9^. The other prevalent theory is that HA is not formed initially, but rather a precursive salt is precipitated, which later transforms into HA, with amorphous calcium carbonate (ACC)^10^, amorphous calcium phosphate (ACP)^11^, and polymer-induced liquid precursors^12^ being the most cited.

A specific quandary faced by the HO community is the sheer quantity of material required for the rapid development of dense bone. For example, the phosphorous concentration in dense HO is estimated to be around 5 mmol cm^−3^ ^13^, slightly higher than in physiological bone (4.2 mmol cm^−3^ ^14^); however, the phosphorous concentration in serum is only 0.001 mmol cm^−3^ ^15^. This has led researchers to seek a specific source of phosphate, for example, dense granules in platelets, which have a high phosphate concentration of around 0.13 mmol cm^−3^ ^16^. Such granules are known to aggregate around bone defects^17^ and, in addition to inorganic polyphosphate^18^, contain high levels of adenosine tri- and di-phosphate (ATP and ADP)^19^. ATP has been shown to be important in HO formation, where remote hydrolysis of this molecule has been shown to mitigate ectopic bone deposition through inhibition of the SMAD 1/5/8 pathway^20^. The large amount of energy released through phosphate cleavage may also be important as, in addition to the materials of formation, bone development also requires a large amount of energy^21^.

In this study, the chemical architecture of human HO was mapped, where the presence of microregions of calcium and no phosphate suggests formation through a precursor species, most likely amorphous calcium carbonate. The transformation of ACC in physiological conditions was then studied, both in the presence and absence of ATP, in order to present a possible mechanism for the chemical development of HO.

## Results

### Chemical composition of HO

**Fig. 1:**
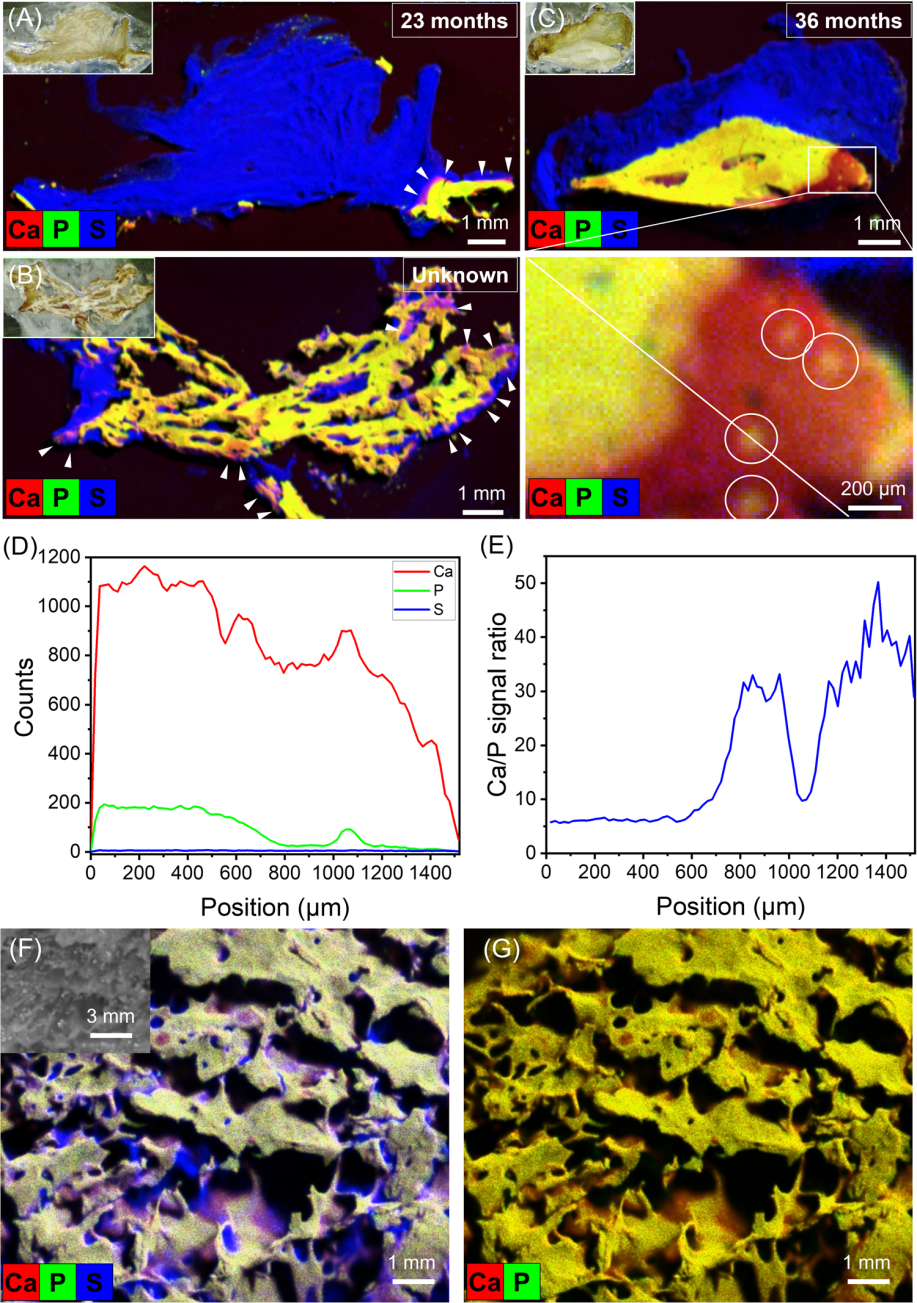
Chemical mapping of HO sections. XRF maps of resin-embedded slices of human HO excised at **A** 23 months, **B** an unknown time and **C** 36 months post-injury. The inset in (**C**) is a zoomed-in area, displaying a largely phosphate-free area with nodules of colocalised calcium and phosphate (circled), where a line scan has been taken to quantify the relative levels of calcium, phosphate and sulphur (**D**), and the ratio of calcium and phosphate (**E**). For comparison, XRF scans of physiological bone are shown in (**F**) and (**G**). Red = calcium; green = phosphorus; blue = sulphur (representing collagen); pink = colocalisation of calcium and sulphur; yellow = colocalisation of calcium and phosphorus (representing calcium phosphate). Insets are corresponding optical micrographs of the HO slices.

The chemical composition of human HO was mapped through XRF scanning. Much of the excised section was unmineralised soft tissue, evidenced by the high sulphur signal (blue), indicating the presence of cysteine-containing proteins such as collagen, and low signals for calcium (red) and phosphorus (green) (Fig. 1A–C). Regions of colocalised calcium and phosphate (yellow) indicated the presence of a calcium phosphate salt, which may be amorphous or crystalline. However, there were also regions with a high calcium but low phosphorous signal, seen at the mineralisation front in Fig. 1A (arrows), at the peripheries in Fig. 1B (arrows), and in a large area in Fig. 1C. Within this large region of phosphate deficiency, there were clear islands of colocalisation (Fig. 1C, insert). A line scan across this region showed a near-constant sulphur signal, a phosphorous signal that was much higher in regions of colocalisation, and a calcium signal that, while always high, was higher in regions of colocalisation (Fig. 1D). The ratio of calcium to phosphorous was also much lower in regions of colocalisation (Fig. 1E). In contrast, XRF maps of mature physiological bone appeared to show colocalisation of calcium and phosphorous throughout, demonstrating significantly more homogeneity than the pathological tissue (Fig. 1F, G).

Given that calcium metal is highly unstable in vivo, there must be a counter-ion that cannot be detected through XRF in these areas, the most likely choice being a carbon species. Further, as amorphous species are more easily directly substituted^22^, and it has been implicated previously in bone mineralisation^22,23^, the phosphate-deficient phase was assumed most likely to be an amorphous calcium carbonate (ACC). ACC is highly unstable in water, rapidly crystallising into vaterite and/or calcite. However, it can be stabilised by many species found in vivo, such as Mg^2+^ ions and ATP. The transition between stabilised ACC and HA was thus studied as a possible chemical mechanism of HO, and thus also physiological bone formation and an explanation for the chemical heterogeneity seen in forming HO.

### Transformation of Mg stabilised ACC

**Fig. 2:**
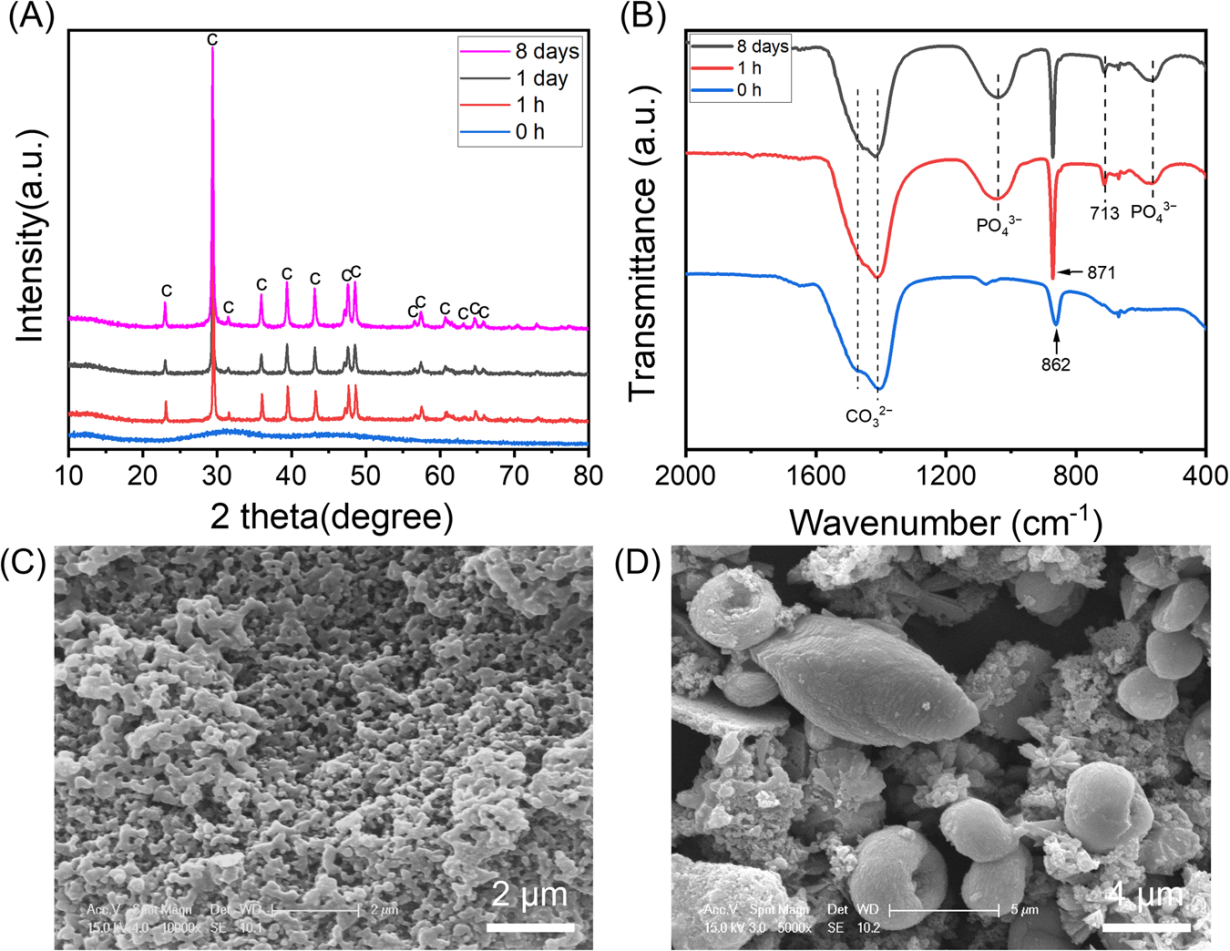
Transformation of Mg stabilised ACC. XRD patterns (**A**) and FTIR spectra (**B**) of ACC stabilised with magnesium before and after submersion in 0.1X PBS at 37 °C. SEM images of the initial ACC material (**C**) and the material formed post-immersion for 8 days (**D**). c, calcite.

ACC was first synthesised and stabilised with Mg^2+^ ions^24,25^. When submerged in a solution containing a physiological level of phosphate, Mg stabilised ACC fully crystallised into calcite after 8 days, though crystallinity was evident through XRD after only 1 h (Fig. 2A). The FTIR spectra of the starting material initially showed a broad carbonate double peak, and a peak at 862 cm^−1^, indicative of ACC (Fig. 2B). Post-immersion, the carbonate peak remained, though it had sharpened and the doublet was less clear, and the peak at 862 cm^−1^ had shifted to a sharp peak at 871 cm^−1^ which, along with the new peak at 713 cm^−1^, indicated calcite formation. Additionally, phosphate peaks could be seen in the post-immersion FTIR spectra; however, no crystalline phosphate salts were detected through XRD. This may indicate that the phosphate salt was amorphous or present in low quantities on the surface of the calcite mineral, giving it an amplified signal in FTIR, a primarily surface technique. SEM images of the material pre- and post-immersion were also taken, showing the transition from associated nanoparticles (Fig. 2C) to what appears to be several phases (Fig. 2D).

### Transformation of ATP stabilised ACC

**Fig. 3:**
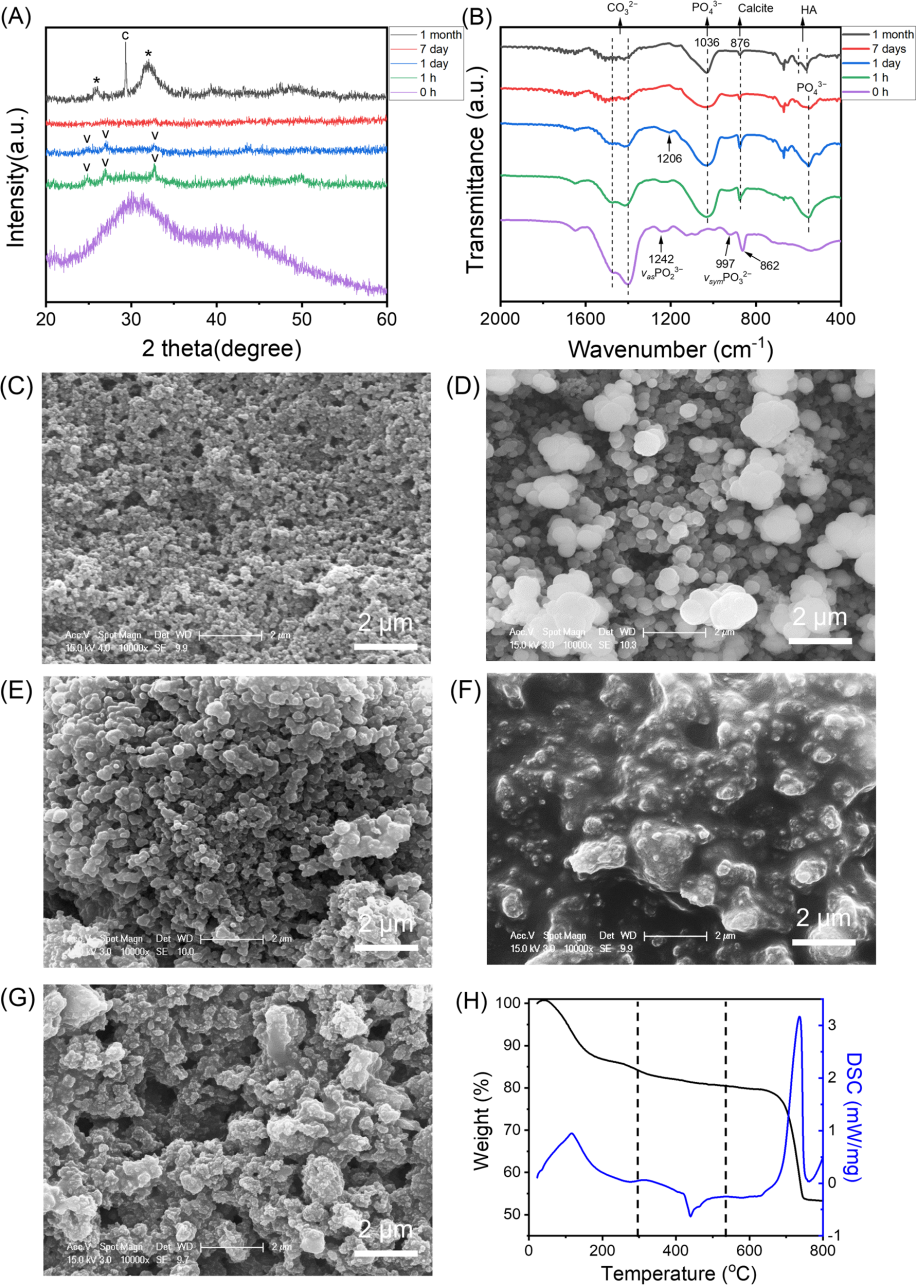
Transformation of ATP stabilised ACC. XRD patterns (**A**) and FTIR spectra (**B**) of ACC stabilised with ATP before and after submersion in 0.1X PBS at 37 °C. SEM images of the initial ACC material (**C**) and the material formed after immersion for 1 h (**D**), 1 day (**E**), 1 week (**F**) and 1 month (**G**). DSC and TGA curves for ATP stabilised ACC powder (**H**). v, vaterite; c, calcite; *, apatite.

ACC can also be stabilised with ATP. At the concentrations studied, ATP proved a more effective stabiliser than magnesium, with full crystallisation occurring between a week and a month of submersion, compared to an hour (Fig. 3A). The transformation here also appeared more complex, with small amounts of vaterite appearing to form from the amorphous powder within 24 h, which had disappeared by a week of incubation, to be replaced by mainly HA and a small proportion of calcite within a month. Further, in the ATP stabilised system, the broad carbonate double peak disappeared from the FTIR spectra within a week, contrary to the Mg stabilised system, and the phosphate peaks, which remained rounded in the Mg stabilised ACC, became notably sharp at 1 month (Fig. 3B). The emergence of PO_4_^3^^−^ absorption bands at 1036, 554 cm^−1^ and the weakened vaterite peaks at 1470, 1402, 876 cm^−1^ after immersion for 7 days, indicated the conversion of ACP from ACC, while the sharper band at 1036 cm^−1^, the split bands of 603 and 564 cm^−1^ and the decreased CO_3_^2−^ bands at 1470 and 1402 cm^−1^ after submersion for 1 month indicated a highly ordered HA crystal structure was eventually formed from ACC^26,27^. The disappearance of the symmetric PO_3_^2−^ and asymmetric PO_2_^3−^ peaks seen in the starting material at 997 and 1242 cm^−1^, respectively, suggested hydrolysis of the phosphate moieties from ATP^16^.

This process was also visualised through SEM imaging, where a similar association of nanoparticles as in the Mg stabilised system can be seen in the ATP stabilised ACC (Fig. 3C). After 1 h, larger, distinct spheres, more crystalline in appearance form (Fig. 3D), which then become smaller and more connected, similar to the amorphous starting material, after 1 day (Fig. 3E), and become entirely indistinct after a week, forming an almost fluid appearance (Fig. 3F). The final form after 1 month appears to be an aggregate of particles (Fig. 3G). The composition of the initial material was assessed through TGA and DSC, which showed three distinct weight-loss phases: weight loss before 200 °C, with a highly exothermic heat flow peak at around 100 °C, suggested loss of free water; the weight lost between 200 and 450 °C, associated with a slightly endothermic heat flow, was assigned to the breakdown of ATP, and the large decrease in weight, with a large exothermic heat flow peak, after 550 °C was likely the decomposition of calcium carbonate to calcium oxide and carbon dioxide^28^ (Fig. 3H).

In order to study the conversion from CaCO_3_ to HA in a shorter time, the system was investigated at an elevated temperature (60 °C) to both accelerate the crystallisation of ACC and the conversion of ACC to calcium phosphate (CaP), mimicking the trend of conversion at 37 °C. By heating to 60 °C, ATP stabilised ACC was converted to HA in only 2 days, compared to >7 days at 37 °C, following the same phase transition albeit with a small amount of vaterite remaining at the reaction’s conclusion (Supplementary Fig. S1A, B). This system was also used to study ATP hydrolysis, and it was found that around 18% of the ATP was entirely released immediately, followed by a linear release up to 60% at 15 h, which tailed off until 90% of the loaded adenosine was released at 48 h (Supplementary Fig. S1D). The initial fast release may be attributed to the free ATP in the sample. Mg stabilised ACC dispersed in 0.1X PBS and 0.15X PBS at 60 °C for 1 and 2 days both exhibited crystalline CaCO_3_ as shown in Supplementary Fig. S2A, B. The 0.15X PBS applied here as a dispersant aims to maintain the same P concentration with ATP-ACC in 0.1X PBS, considering the hydrolysis of ATP. Vaterite and calcite microparticles were formed, as shown in the SEM image (Supplementary Fig. S2C), and EDX mapping and EDX spectrum in Supplementary Fig. S2D, E demonstrate the formation of CaP nanoparticles and crystalline CaCO_3_ on the microscale. The EDX mapping indicates that CaP formed on the surface of crystalline CaCO_3_ via a dissolution-precipitation reaction.

**Fig. 4:**
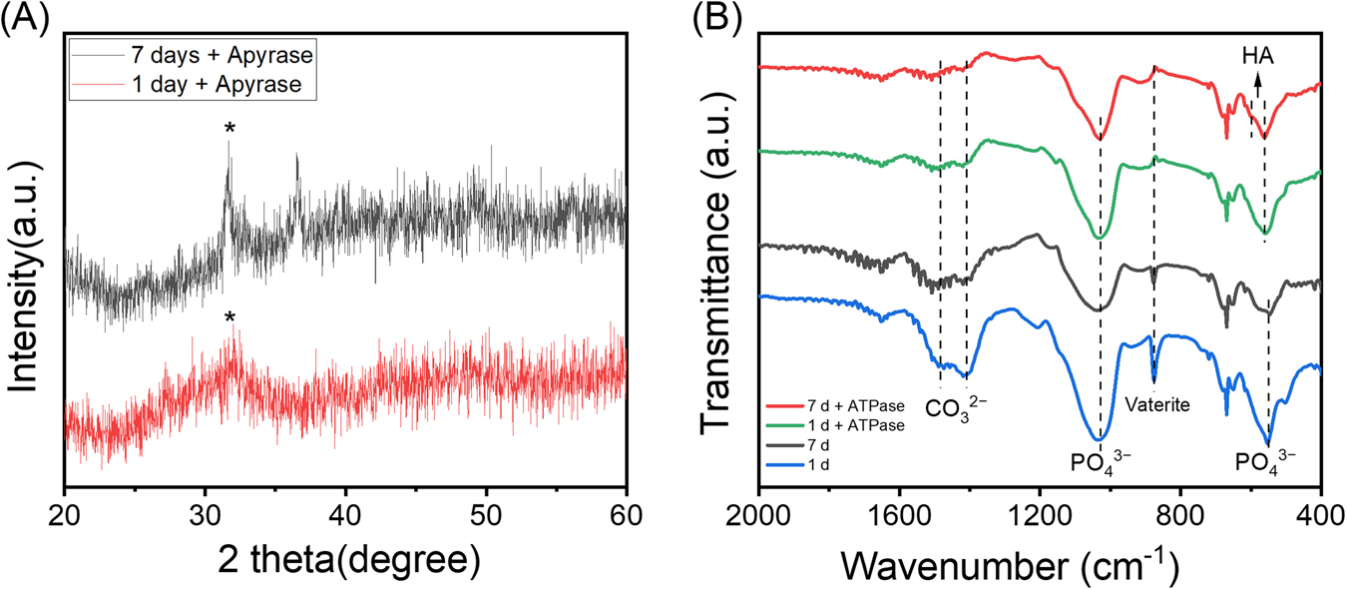
Transformation in the presence of apyrase. XRD patterns (**A**) and FTIR spectra (**B**) of ATP stabilised ACC submerged in 0.1X PBS for 1 or 7 days at 37 °C, before and after the addition of apyrase incubated for another day. *, apatite.

To examine how this process might be accelerated in vivo, the ATP-stabilised ACC system was submerged in 0.1X PBS for 1 or 7 days, as previously, before the addition of the enzyme apyrase and then incubated for another day to accelerate the decomposition of ATP. The addition of apyrase appeared to hasten the crystallisation to HA to 7 days (Fig. 4A), compared to 1 month in the absence of apyrase (Fig. 3A). This can be seen more clearly in the FTIR spectra, where 1 day in the presence of apyrase appeared to entirely remove both the broad carbonate peak and the peak corresponding to crystalline vaterite, and sharpen the phosphate peak (Fig. 4B). The FTIR spectrum for the sample soaked for 7 days shows the formation of highly ordered HA crystals^26,27^.

### Transformation of ACC in high phosphate concentration (0.1 M)

**Fig. 5:**
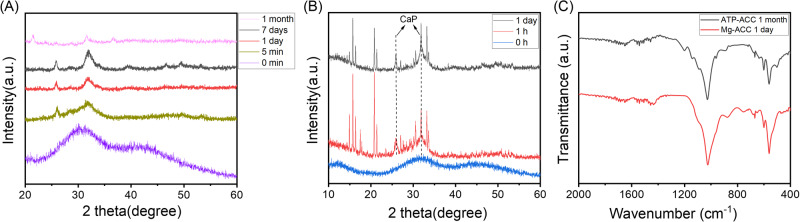
Transformation in ammonium phosphate. XRD patterns showing the changes in ATP stabilised ACC (**A**) and Mg^2+^ stabilised ACC (**B**) when submerged in 0.1 M (NH_4_)_2_HPO_4_ solution at 37 °C; FTIR spectra (**C**) of both ACC samples treated in 0.1 M (NH_4_)_2_HPO_4_.

Finally, to understand the role of environmental phosphate concentration, both the ATP and Mg stabilised ACC sample powders were submerged in 0.1 M (NH_4_)_2_HPO_4_ solution, which has 100 times the phosphate concentration of 0.1X PBS. The ATP stabilised system formed HA much more rapidly than when submerged in 0.1X PBS, with HA peaks visible after only 5 min and clear crystallinity within a day (Fig. 5A). The Mg stabilised system, contrary to when immersed in 0.1X PBS, also formed HA in 0.1 M (NH_4_)_2_HPO_4_, with additional peaks visible due to the formation of MgNH_4_PO_4_ (Fig. 5B). The FTIR spectra in Fig. 5C indicate the formation of HA crystals^26,27^.

## Discussion

Chemical mapping of human HO tissue using XRF revealed numerous regions which were calcium-rich but contained no detectable phosphate (Fig. 1). This is in contrast with the mapping of mature physiological bone, where the calcium and phosphate were localised throughout the majority of the sample. Indeed, although there were some small areas that were calcium-rich-phosphate-poor in normal bone, the mineral composition was generally much more homogeneous, suggesting that the mineral phase in the tissue was predominantly calcium phosphate, as would be expected for human bone. The areas with no phosphate may be undergoing remodelling and mineralisation, or this may be an artefact due to surface roughness. While any element lighter than neon cannot be categorically ruled out through XRF, the most likely light counter-ion to calcium in vivo is a carbon species. Again, while this is possible to be any number of carbon species, such as citrate, lactate or oxalate, carbonate is perhaps the most likely, as many forms of calcium carbonate are conserved throughout the animal kingdom^29^, and ACC has been implicated as a bone mineralisation precursor for decades^22,23^.

The conversion of ACC in physiological solutions was therefore studied. ACC is highly unstable in water^30^ and must therefore be stabilised by some species to exist in vivo. Many additives, including Mg^2+^ ions^31,32^, phosphorus-containing compounds^28^, biomolecules^33^ and polymers^34^ have been shown to form stable ACC during its formation; two such species found in vivo are Mg^2+^ ions and polyphosphates such as ATP^16,24^. Mg^2+^ was studied first, and at the concentration studied, ACC appeared to be stable for up to 1 h in 0.1X PBS, which contains the same phosphate concentration as serum, at 37 °C (Fig. 2). However, on crystallisation, Mg stabilised ACC primarily transformed into a calcium carbonate mineral, calcite, rather than the HA that would be expected in bone. A phosphate phase did appear to form alongside the calcite, though crucially, it was not incorporated into the carbonate phase and formed separately. As no crystalline phosphate phase was detected through XRD, it is likely that this phase was amorphous and/or formed only in small amounts on the calcite surface. This hypothesis is supported by the EDX imaging in Supplementary Fig. S2D, showing the phosphate phase forming a layer on top of the calcite mineral. This amorphous phase may crystallise over long time periods. Regardless, the short-term stabilisation and final formation of mainly calcite, a mineral not found in the human body, suggest that this is, at least principally, not the process that occurs in human bone formation.

ATP is found ubiquitously in vivo and has also been shown to stabilise ACC in a concentration-dependent manner; up to 12 days in PBS, at higher ATP concentrations than those used in this study^28,33^. The concentration used here, 1 mM, was higher than normally found in serum; however, local concentrations of ATP can reach 3–5 mM with the accumulation and activation of platelets following trauma^35^. Interestingly, following submersion, some vaterite crystallisation occurred within 1 h; however, this appeared to resolubilise with the formation of an amorphous phase, with the final apatite mineral forming between 7 days and 1 month (Fig. 3). The significant delay in crystallisation, and the final product being HA, make ATP a more likely stabiliser of ACC in vivo that Mg. Of particular interest was the formation of the X-ray amorphous phase at 7 days (Fig. 3A), which suggested that the pathway of HA formation underwent a metastable precursor rather than direct ionic substitution from vaterite. While direct substitution is possible in vaterite, but notably not the thermodynamically stable calcite formed in the Mg stabilised system, this process is far more favourable in ACC^22^. Further, the lack of carbonate peaks suggested that this amorphous phase was not simply the reprecipitation of ACC, but a different phase, most probably ACP. Indeed, a study synthesising HA from ACC in non-physiological conditions found that this reaction progressed *via* ACP^36^. This is an exciting finding, as ACP is also commonly implicated as a precursor in human bone mineralisation^37^.

As HO is commonly defined as bone formation in soft tissues, and thus HO and bone are identical, at least at the microscale^2^, it was assumed that HA would be the final mineral produced in both cases. However, interestingly, a calcium non-phosphate mineral appeared in the samples tested up to 3 years post-injury. This is long after HO is considered metabolically ‘mature’, usually between 6 months and 1 year, though this can depend on multiple factors^38^. Indeed, it has been shown that HO can continue to remodel after 3 years^39^. It may be that the bone in this particular patient was still indeed maturing, or else that the non-phosphatic calcium species is stable and persists indefinitely. Due to differing formation pathways, it may be that HO and skeletal bone are chemically distinct. Indeed, studies that examine HO tend to focus on clinical and morphological aspects, relying on radiographic, microscopic and histological examination, techniques incapable of probing the mineral chemistry^7,39^. Further studies examining the chemistry of HO, including further XRF studies, XRD analyses and Raman spectroscopy, are required to confirm or refute this hypothesis.

The conversion of ACC to calcite or HA could be thermodynamically driven, although it is still unclear whether the conversion to HA undergoes an internal structural bulk rearrangement of the metastable precursor or a dissolution-reprecipitation process^40^. The reappearance of an amorphous phase at day 7 may indicate the latter in this case (Fig. 3A). The fate of ACC during crystallisation may be determined by the priorities between its conversion to calcite and CaP; this process is likely governed by the more kosmotropic phosphate ions and polyphosphate molecules, relative to the lighter and more chaotropic Ca^2+^ ions acting as diffusive fillers in the phosphate network^41^. In the ATP-stabilised ACC system, the incorporation of phosphate and gradual formation of phosphate framework within the ACC precursor happened before the crystallisation of ACC into stable calcite. A similar phenomenon has also been reported for peptides rich in aspartic acid and glutamic acid, which accelerate the conversion of ACC into HA at phosphate concentrations (50 and 500 mM) much higher than physiological conditions^22^. The crystallisation of ACC into calcite may take the dominant role in the Mg system due to the poor stability of Mg-stabilised ACC, leading to crystallisation before phosphate incorporation.

In vivo, of course, there are many enzymes capable of breaking down ATP, and it is far more likely that it is broken down in this way than by thermal hydrolysis. These include enzymes, such as apyrase and tissue non-specific alkaline phosphatases, that cleave one phosphate unit sequentially from ATP, ADP and AMP, resulting in adenosine and three orthophosphate units per ATP molecule^42–44^. Enzymes also exist in vivo, such as ectonucleotide pyrophosphatase/phosphodiesterase 1, that cleave two phosphate units, producing pyrophosphate and AMP. Pyrophosphate is a known inhibitor of mineralisation in the body and has also been shown to stabilise ACC^45^. However, pyrophosphate can also be degraded enzymatically into two orthophosphate units, simultaneously removing a mineralisation inhibitor and producing material for apatite formation^21^. Other phosphatic compounds found in vivo, such as inorganic polyphosphates, which have also been shown to stabilise ACC, may act similarly^46^.

**Fig. 6:**
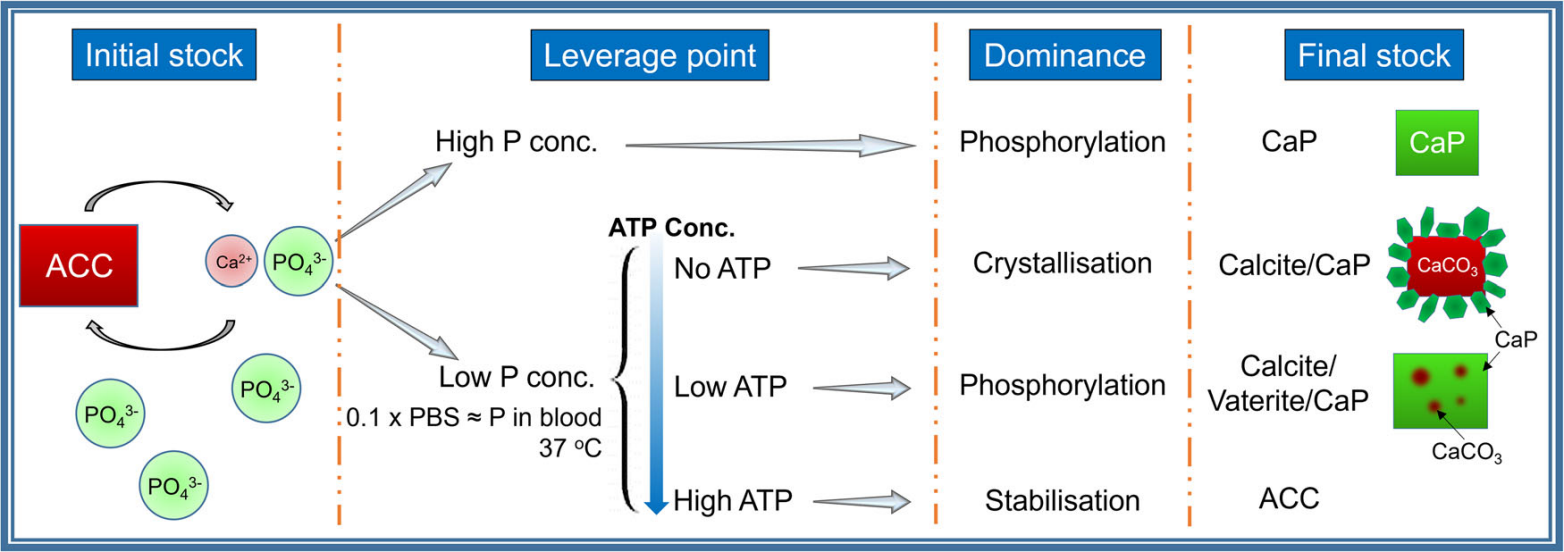
Summary of the phase transitions of ACC in different media. When subject to a high phosphate concentration, ACC transforms into apatite, regardless of stabilisation. At a physiological phosphate concentration, in the absence of ATP stabilisation, ACC transforms rapidly into calcite, with perhaps a small amount of surface phosphate. At high ATP concentrations, ACC is stable for long time periods; however, at low concentrations, the breakdown of ATP yields the formation of apatite.

To examine the effect of phosphatase enzymes, apyrase was added to the system, and indeed far more rapid crystallisation into HA was observed (Fig. 4). However, the important implication of this is the possibility of localised transition through enzymatic activity: the initial ACC laydown is stabilised by ATP then when locally broken down by enzymatic activity of cells, the dual action of removal of ATP stabilisation and release of phosphate triggers local transition from ACC to HA. This might be a reasonable mechanistic explanation for the nodular colocalisation of calcium and phosphate within a phosphate-depleted region of HO (Fig. 1C). Clearly localisation of this effect is important, as a high concentration of phosphate triggers rapid phase transformation of ACC to apatite regardless of stabilisation (Fig. 5). The key factors affect the transformations of ACC occurred under different conditions in the ATP-ACC conversion system are summarised in Fig. 6.

## Conclusions

ACC was stabilised by ATP in a physiological solution, and the incorporation of different contents of ATP significantly affected its conversion pathway. In this study, the transformation followed an ACC → ACP → HA pathway after submersion in a physiological phosphate solution for 1 month. The transformation process can be triggered by the breakdown of ATP, resulting in HA formation, suggesting that a carbonate-based precursor could reasonably exist in HO and, by extension, form physiological bone. The breakdown of ATP can be triggered by enzymes, both removing its stabilising effect and increasing the local phosphate concentration. Local enzymatic degradation of ATP through cellular activity could lead to the apatitic nodules seen in the otherwise phosphate-deficient regions of HO.

## Methods

### Materials

Calcium chloride dihydrate was purchased from G-Biosciences (St Louis, MO, USA). Sodium carbonate decahydrate was bought from Alfa Aesar (Lancashire, UK). Magnesium chloride hexahydrate, adenosine 5’-triphosphate (ATP) disodium salt hydrate, Apyrase from potatoes, Dulbecco’s phosphate buffered saline (PBS) with calcium chloride and magnesium chloride was ordered from Sigma-Aldrich Company Ltd. Ammonium phosphate dibasic was obtained from Fluka (Germany). Optimal cutting temperature (OCT) compound was supplied by VWR Chemicals (Leuven, Belgium). All of the chemicals were used without further purification.

### HO tissue

Human HO tissue was acquired from the Human Biomaterials Resource Centre at the University of Birmingham, UK, the details of which are listed in Supplementary Table S1. The HO samples were defrosted from −80 **°**C and embedded in optimal cutting temperature (OCT) compound under vacuum conditions (20 kPa). The embedded samples were then sectioned into 100-μm thickness slices in a cryostat using a Leica microtome with a tungsten carbide blade. The resulting slices were air-dried on glass slides.

The mature physiological bone block (thickness: <1 cm) was obtained from a human femur, which was cut to create a macroscopically flat surface to image. The bone block was not embedded in resin in order to minimise the background effects of the chemical preparation. Tissue transfer and handling were conducted under the approval of the National Research Ethics Service (15/NW/0079) and in accordance with the Human Tissue Act 2004.

### Amorphous calcium carbonate (ACC) synthesis

ACC was fabricated by combining equal volumes of equal molarity of CaCl_2_ and Na_2_CO_3_ solutions^25^. To prepare Mg-stabilised ACC, CaCl_2_ (0.1 M, 200 mL), Na_2_CO_3_ (0.1 M, 200 mL) and MgCl_2_ (0.5 M, 40 mL) solutions were combined through vigorous magnetic stirring for 15 s, with no pH adjustment. Following precipitation, the suspension was centrifuged (1 min at 4000 rpm), and the supernatant fluid was removed to recover the particles. These were washed thrice with absolute ethanol and vacuum dried for 12 h to yield a dry powder. The same procedure was followed to produce the ATP-stabilised ACC, but the initial CaCl_2_ and Na_2_CO_3_ solutions were at a concentration of 25 mM, and no MgCl_2_ solution was added; instead, 1 mM of ATP was added to the CaCl_2_ solution prior to combining with the Na_2_CO_3_ solution.

### Transformation of ACC in phosphate solutions

ACC powders were dispersed in 40 mL of solution (0.1X PBS, 0.15X PBS or 0.1 M (NH_4_)_2_HPO_4_) at a concentration of 0.5 mg mL^−1^, under magnetic stirring at 250 rpm at 37 or 60 °C. Aliquots were taken at each time point and recovered by centrifugation and washing with ethanol. To study the effects of enzymatic degradation, apyrase (70 units) was added for 24 h, after either 1 day or 7 days, to ACC powders (20 mg) immersed in 0.1X PBS (40 mL).

### Physicochemical characterisation of powders

The crystallinity and phase composition of the materials were determined by powder X-ray diffraction (XRD) using a D8 Advance (Bruker) X-ray diffractometer with Cu-Ka radiation (l = 1.5406 Å). Fourier-transform infrared (FR-IR) spectra were obtained using a Tensor 27 (Bruker) spectrometer, measured from 4000 to 400 cm^−1^. Peak assignments were obtained from^26,27,47–49^. Scanning electron microscopy (SEM) images were attained using a TM3030 (Hitachi), in secondary electron mode, with an accelerating voltage of 15 kV. Thermogravimetric analysis (TGA) and differential scanning calorimetry (DSC) measurements were carried out on a STA 449 F3 system (Netzsch) with a heating rate of 10 K min^−1^ from room temperature to 800 °C in a nitrogen flow.

### X-ray fluorescence

The chemical composition of the HO sections was mapped using a Tornado M4 micro X-ray fluorescence (XRF) system (Bruker Nano Gmbh, Berlin, Germany) equipped with a rhodium X-ray tube operating at 50 kV and 400 μA, a 25-μm spot size, 20 μm spot distance, 60 ms exposure time and operated at a reduced pressure of 50 mbar. Note that XRF can only detect elements heavier than neon.

### Release of adenosine

To measure the formation and release of adenosines, including ATP and its dephosphorylated forms, ATP-ACC (50 mg) was placed into dialysis tubing and transferred into 0.1X PBS (100 mL) at 60 °C under mild agitation (250 rpm). The released sample aliquots (2 mL) were collected at time points between 5 min and 48 h, with the replacement of fresh 0.1X PBS (2 mL). The concentration of adenosines in each aliquot was found by measuring the absorbance at a wavelength of 258 nm (Cecil 2021 UV spectrophotometer) and then finding the concentration from a standard curve of known adenosine concentration. The original ATP content in the sample was found by immersing the sample (10 mg) in 0.1 M HCl (5 mL) overnight to completely dissolve the ACC and measuring its UV absorbance. The payload of ATP in ATP-ACC was calculated to be 16.73% based on the below equation.

$${{{{{\rm{Payload}}}}}}=\frac{{{{{{\rm{Mass}}}}}}\; {{{{{\rm{of}}}}}}\; {{{{{\rm{ATP}}}}}}\; {{{{{\rm{recovered}}}}}}\; {{{{{\rm{from}}}}}}\; {{{{{\rm{the}}}}}}\; {{{{{\rm{obtained}}}}}}\; {{{{{\rm{ATP}}}}}}\mbox{-}{{{{{\rm{ACC}}}}}}\; {{{{{\rm{powder}}}}}}}{{{{{{\rm{Mass}}}}}}\; {{{{{\rm{of}}}}}}\; {{{{{\rm{ATP}}}}}}\mbox{-}{{{{{\rm{ACC}}}}}}\; {{{{{\rm{powder}}}}}}}$$

All of the experiments in the paper were repeated thrice.

### Supplementary information

Supplementary Information

## Data Availability

The authors declare that the data supporting the findings of this study are available within the paper and its supplementary information files. Should any raw data files be needed in another format, they are available from the corresponding author upon reasonable request.
