# Supplementary material for: Triggered metabolism of adenosine triphosphate as an explanation for the chemical heterogeneity of heterotopic ossification
Source: Commun Chem. 2023 Oct 19;6:227. doi: 10.1038/s42004-023-01015-z (PMC10587346; doi:10.1038/s42004-023-01015-z)
Supplement: Supplementary file 1 — Supplementary Information [file 42004_2023_1015_MOESM1_ESM.pdf]

Supplementary Information

<u>HO sample details</u>

Table S1. Clinical details of HO samples from patients suffering from HO used in this research. IED = Improvised explosive device. The abbreviated clinical data is to ensure confidentiality.

Patient case	Age (year)	Gender	Mechanism	Anatomical Site	Interval Between Injury and Excision
1	30	Male	IED Blast	Right lower limb residuum	23 months
2	36	Male	Fall from height	Not recorded	No data
3	42	Male	IED Blast	Left lower limb stump	36 months

<u>Conversion at elevated temperature</u>

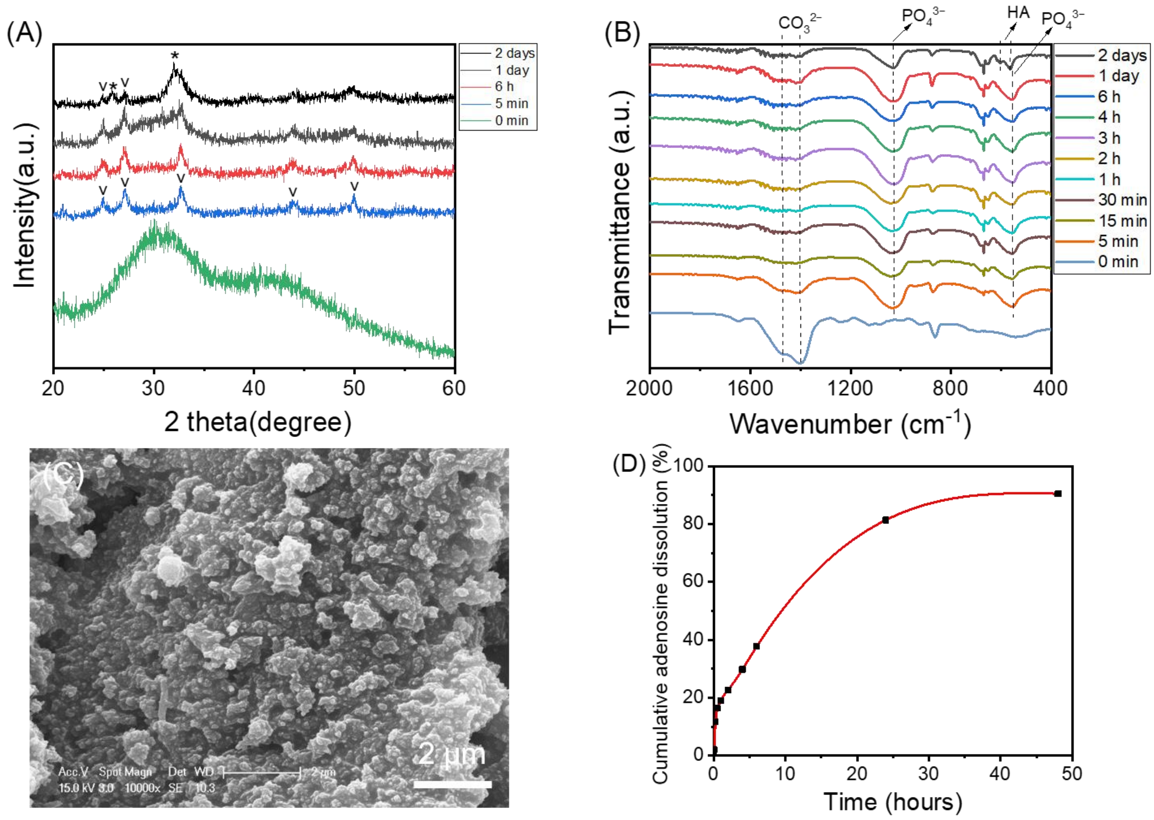

Figure S1: Transformation of ATP stabilised ACC at elevated temperatures. *XRD patterns (A) and FTIR spectra (B) of ACC stabilised with ATP before and after submersion in 0.1X PBS at 60 °C. SEM image of the material formed after immersion in 0.1X PBS for 2 days at 60 °C (C). Graph of adenosine released over the 2 days of immersion.*

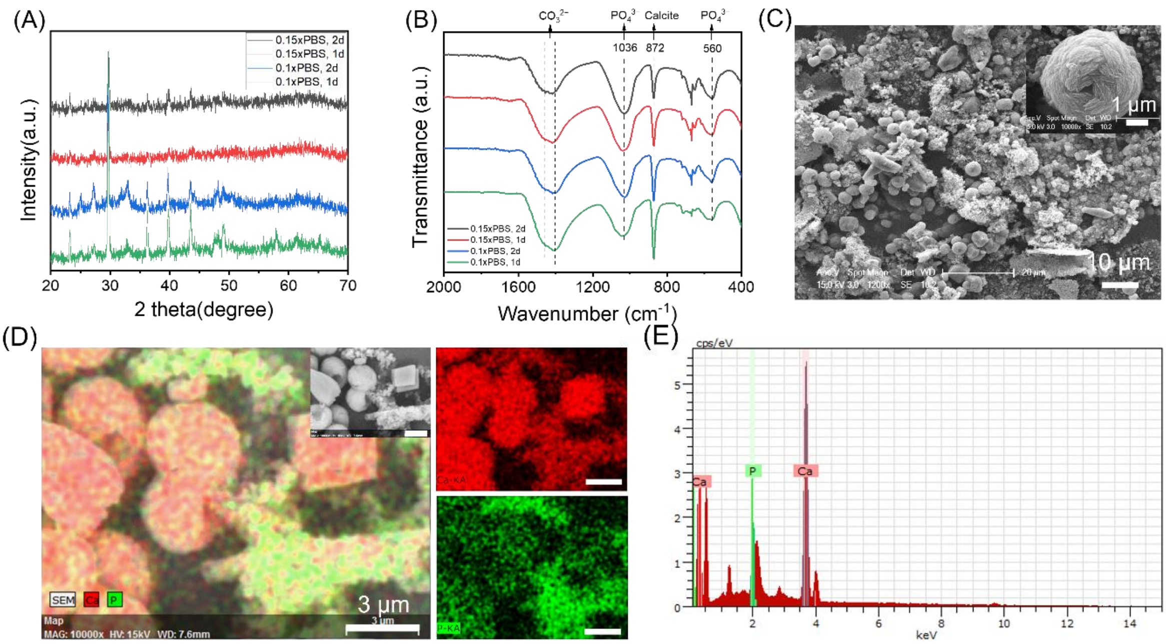

Figure S2:Transformation of Mg stabilised ACC at elevated temperatures. *XRD patterns (A) and FTIR spectra (B) of ACC stabilised with Mg before and after submersion in 0.1X and 0.15X PBS at 60 °C. SEM image (C), EDX mapping (D) and EDX spectrum (E) of the material formed after immersion in 0.1X PBS for 2 days at 60 °C.*
